# Soft X-ray magnetic scattering studies of 3D magnetic morphology along buried interfaces in NiFe/CoPd/NiFe nanostructures

**DOI:** 10.1038/s41598-019-51098-9

**Published:** 2019-10-15

**Authors:** Samuel Flewett, Thiago J. A. Mori, Alexandra Ovalle, Simón Oyarzún, Antonio Ibáñez, Sebastián Michea, Juan Escrig, Juliano Denardin

**Affiliations:** 1 Instituto de Física, Pontificia Universidad Católica de Valparaíso, Avenida Universidad 330, Valparaíso, Chile; 2 Laboratório Nacional de Luz Síncrotron, Centro Nacional de Pesquisa em Energia e Materiais, Campinas, SP 13083-970 Brazil; 3 Departamento de Física, CEDENNA,, Universidad de Santiago de Chile, USACH, Av. Ecuador, 3493 Santiago, Chile; 4 Instituto de Ciencias Químicas Aplicadas. Facultad de Ingeniería. Universidad Autónoma de Chile. Av. El Llano Subercaseaux, 2801 San Miguel, Chile

**Keywords:** Magnetic properties and materials, Magnetic properties and materials, Magneto-optics, X-rays

## Abstract

With the continuing interest in new magnetic materials for sensor devices and data storage applications, the community needs reliable and sensitive tools for the characterization of such materials. Soft X-rays tuned to elemental absorption edges are a depth and element sensitive probe of magnetic structure at the nanoscale, and scattering measurements have the potential to provide 3D magnetic structural information of the material. In this work we develop a methodology in transmission geometry that allows one to probe the spatial distribution of the magnetization along the different layers of magnetic heterostructures. We study the in-plane/out-of-plane transition of magnetic domains in multilayer thin film systems consisting of two layers of NiFe top and bottom, and a 50 repeat Co/Pd multilayer in the centre. The experimental data are analysed by simulating scattering data starting from micromagnetic simulations, and we find that the out of plane domains of the Co/Pd multilayer intrude into the NiFe layers to a greater extent than would be expected from micromagnetic simulations performed using the standard magnetically isotropic input parameters for the NiFe layers.

## Introduction

The continuous research interest into magnetism along with the development of new devices has been possible mainly due to both the continuous discovery of new classes of materials and the development of techniques for characterizing such materials. This article serves two primary purposes. On the one hand, to study the interface between the perpendicular anisotropy exhibited by the Co/Pd multilayer samples versus the roughly isotropic behaviour of the NiFe layers. Secondly, to test the sensitivity of transmission geometry X-ray resonant magnetic scattering (XRMS) applied to the 3D characterization of such samples with buried interfaces.

In the last decades, the advancement of methods for growing, nano-patterning and characterizing the properties of thin films has led to the development of interesting magnetic structures at the nanoscale^1,2^. Consequently, phenomena of potential technological interest arise at surfaces and interfaces of such magnetic nanostructures. Some of the materials of interest for the development of modern magnetic memory devices, for instance, present stripe-like magnetic domains structures in the remanent state^3–6^, which arise due to the presence of strong perpendicular magnetic anisotropy (PMA). In turn, the occurrence of PMA together with the interfacial Dzyaloshinskii-Moriya interaction (iDMI) in multilayers can give rise to promising chiral structures such as magnetic bubbles or skyrmions^7–9^. Recently, it has been demonstrated that magnetic bubbles in a PMA multilayer can be imprinted into an adjacent soft magnetic layer with in-plane anisotropy, leading to strong modifications of the high frequency magnetization dynamics of the structure^10^. In sum, magnetic interactions and anisotropies present a relationship with the formation processes of the magnetic domains and domain walls and are strongly influenced by the interfaces in heterostructure thin films. In this sense, the knowledge of the physics behind magnetic domains and domain walls is primordial to the understanding of the mechanisms associated with magnetic interactions in buried interfaces and, from an applications viewpoint, to design devices that can be used in sensors and actuators to microwaves, magnetoimpedance sensors, magnetic memories, spintronics, etc.

The use of resonant magnetic scattering in transmission geometry^11–13^ has had relatively little study compared with its reflection counterpart^2,14–20^ (only a small selection of references cited – see the review article^17^ for full details), especially for the aims of 3D characterization where most effort has been focused on scanning and full field microscopy^21–26^, recently including its ptychographic variant^27–30^. Scattering measurements, which allow the statistical properties of a sample to be characterized, have two major distinct advantages over scanning microscopy measurements due to the simpler experimental configuration: (1) the lack of a need to maintain a precisely reproducible sample in real space for each and every angular projection, and (2) the ability to work with lower levels of beam coherence thus allowing a higher photon flux. These advantages should allow the rapid application of this experimental modality to samples in different environment conditions such as varying temperatures and external magnetic fields. Transmission geometry has an advantage over reflection geometry in that it is sensitive to the bulk magnetization pattern, and not just the magnetism probed at the interface of two materials with differing refractive indices. Multilayer samples are well suited to reflection work due to their multiple interfaces which serve as a proxy for the bulk, however, non-multilayer samples such as the NiFe layers studied here do not share this property.

The main challenge inherent with scattering techniques is the need for an often complex modelling scheme to analyze experimental data. In this work, we develop a combined experimental-simulation methodology that allows us to analyse transmission mode XRMS patterns to understand the spatial distribution of the magnetization along the different layers of magnetic heterostructures. Compared with our previous work^11^, the modelling scheme utilized here has been substantially improved, with the previous unphysical model of perpendicularly aligned domains interspersed by triangular closure domains replaced by a continuous magnetization vector field guided by micromagnetic simulations. We additionally increased the complexity of the system under study, studying in this present case trilayer samples of consisting of a 50 repeat (Co 0.8 nm/Pd 0.8 nm) multilayer sandwiched between two layers of either 20 or 40 nm of Ni_0.8_Fe_0.2_, in addition to a simple 50 repeat (Co 0.8 nm/Pd 0.8 nm) multilayer used as a control sample.

The paper is structured as follows: firstly, a section explaining the simulation protocol; and secondly, a section presenting and discussing the experimental results. This is followed by a discussion of the sensitivity of the measurements in terms of differentiating between different candidate magnetisation distributions, and in this section we find evidence which strongly suggests that the out of plane domains of the Co/Pd multilayer intrudes to a greater extent than what would be predicted from micromagnetic simulations using standard input parameters. The experimental procedure is discussed in the final section of the paper.

### Simulations and experimental procedures

Unlike tomography, scattering measurements do not provide sufficient information for an unconstrained sample reconstruction, obliging one to define a parameterized model a-priori to allow the simulation of scattering patterns with varying model parameters. In our previous work, we used a binary out-of-plane vs in-plane triangular closure domain model^11^, and an example of a continuous model which could have been used was published recently in^31^. We opted to use the package OOMMF^32^ as the starting point for our simulations solving the Landau-Lifschitz-Gilbert equations for a magnetic volume and a set of magnetic parameters *A*, *K* and *M*_*s*_. These parameters correspond to the exchange stiffness parameter, the anisotropy constant, and the material saturation magnetization, respectively. To seed the simulations, we characterised each of the samples subject to synchrotron measurement in addition to two control samples consisting of 20 and 40 nm NiFe using a Vibrating Sample Magnetometer (VSM) to extract values of their anisotropy energies and saturation magnetisations. For the complete samples we measured *M*_*s*_ values 6.5 × 10^5^ Am^−1^ for the samples with NiFe present, and 6.8 × 10^5^ Am^−1^ for the control sample consisting of only the Co/Pd multilayer. The *M*_*s*_ values of the NiFe layers deposited individually were measured at 5.4 × 10^5^ Am^−1^ for both the 20 nm and 40 nm films. All values of *Ms* are estimated to have a measurement precision of ±10%. The saturation fields *Hs* for the samples were 7.5 kOe for the pure Co/Pd Multilayer, 7.2 kOe for the sample with 20 nm NiFe layers, and 6.8 kOe for the sample with 40 nm NiFe layers. Using the standard expression $K=\frac{1}{2}{H}_{s}{M}_{s}$, the estimated values of *K* were 2.5 × 10^5^ Jm^−3^ for the pure Co/Pd multilayer, and 2.34 × 10^5^ Jm^−3^ for the sample with 20 nm NiFe layers, and 2.21 × 10^5^ Jm^−3^ for the sample with 40 nm NiFe layers. Assuming that the uncertainties on *Ms* and *Hs* are independent, we estimate the uncertainty on *K* at ±14%. Our estimated values for these parameters measured are close to those reported in the literature^33–38^ for similar classes of samples, and full details of the sample characterisation via VSM including the hysteresis loops can be found in supplementary material.

In the course of performing the simulations, the exchange stiffness parameter remained free to be adjusted according to the measured domain periodicity, and for simplicity the same value of the exchange stiffness was used for both the CoPd stack and the NiFe layers. Tests with differing values of the exchange stiffness in the CoPd versus the NiFe layers (maintaining constant periodicity) did not produce appreciable changes to the calculated X-ray scattering patterns.

For the samples with NiFe present, the modelling of the induced out-of-plane magnetisation in the NiFe layers posed a challenge. It is generally accepted that NiFe is a magnetically soft material which tends to display in-plane magnetisation, and the first candidate for comparison with the experimental measurements assumed an anisotropy of zero in the NiFe layers, and an anisotropy in the CoPd multilayer set such that the overall anisotropy of the entire sample was equal to the value obtained in the VSM. Allowing a non-zero value of *K* of the order of 1 × 10^5^ Jm^−3^ (out of plane) for the NiFe layers, however, produced a superior quality fit to the experimental data. This readjustment of *K* for the NiFe and CoPd layers under the constraint that their weighted average maintain a constant value was therefore the only adjustable parameter used to adjust fit the simulation to the data after tuning the exchange stiffness *A* so as to maintain the measured periodicity. This theme of the induced out-of-plane anisotropy in the NiFe layers will be revisited in the section discussing the experimental sensitivity, where simulated scattering patterns for different choices of *K* are compared. It is however worth noting that in the micromagnetic simulations the input value of *K* is an effective anisotropy, and that there are other factors such as impurities, temperature and stress which are not included in the simulation code and which could explain the discrepancy between the predicted results (with *K* = 0) and those observed experimentally.

The micromagnetic simulations were all performed using the default damping of 0.5 on a 1,000 × 1,000 × *t* nm^3^ 3D grid with 5 × 5 × 5 nm^3^ voxel size and periodic boundary conditions (the thickness *t* depending upon the sample – 80, 120 or 160 nm).

Given the main purpose of the micromagnetic simulations was to generate a candidate spatial magnetisation distribution with which to seed the scattering simulation code, we made some compromises in order to expediate the process. Due to the fact that speed of the micromagnetic solver is non-linear with regards to the size of the magnetic volume, we performed the simulation over a region much smaller than the beam size, and we additionally used a shorter than ideal relaxation time resulting in a residual uncertainty regarding the real magnetic parameters *A* and *K*. From the OOMMF simulation we extracted the average values of the magnetization orientation as a function of perpendicular distance from a domain wall and applied these values to an artificially generated binary domain pattern that shares the size and order parameters of the sample under investigation.

The binary out-of-plane domain pattern is generated using the same recipe as used in our previous work^11^, however, instead of inserting triangular closure domains on the top and bottom surfaces at the domain walls, we tilt the magnetization vector $[{M^{\prime} }_{{\boldsymbol{x}}},\,{M^{\prime} }_{{\boldsymbol{y}}},\,{M^{\prime} }_{{\boldsymbol{z}}}]\,\,$at each point along the locus of points $(d^{\prime} ,\,z^{\prime} )$ corresponding to the distance from the binary domain wall and the depth within the sample *z* respectively according to the following set of equations^31^:

1 $$\begin{array}{c}{{\boldsymbol{M}}}_{{\boldsymbol{x}}}(d^{\prime} ,\,z^{\prime} )=\sqrt{1-{M}_{{\boldsymbol{z}}}{(d,z)}^{2}}\,sin(\alpha (d,\,z)),\\ \,{{\boldsymbol{M}}}_{{\boldsymbol{y}}}(d^{\prime} ,\,z^{\prime} )=\sqrt{1-{M}_{{\boldsymbol{z}}}{(d,z)}^{2}}cos(\alpha ((d,\,z))),\\ \,\,\,{{\boldsymbol{M}}}_{{\boldsymbol{z}}}(d^{\prime} ,\,z^{\prime} )={M}_{{\boldsymbol{z}}}(d,\,z).\end{array}$$

**Figure 1:**
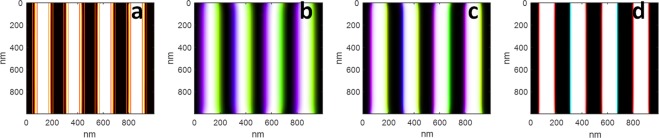
Visualization of the construction of the simulations based on a 1 × 1 μm^2^ thin film micromagnetic simulation (the same as used in Fig. 4). In (**a**) we show a slice of M_z_ through the middle of the sample with the red line marking the locus of points 3 pixels (15 nm) from the domain wall boundary. Values of [Mx(d,z), My(d,z), Mz(d,z)] and α(d, z) are extracted by avereging along all such lines for different values of d.  In (**b**–**d**) we show horizontal slices at different depths in the sample on an HSL colourmap, where the black and white contrast is determined by M_z_, and the colour by the orientation of the in-plane component. In (**b**), the slice is on the surface showing the large Neel type walls present on the surface of the NiFe layer, in (**c**) we show at a depth of 25 nm where smaller Neel walls are present, and in (**d**) we show a slice through the centre of the CoPd layer where small Bloch walls are present.

These equations depend upon the average values of the magnetization generated in the OOMMF pattern $[{M}_{{\boldsymbol{x}}}(d,\,z),\,{M}_{{\boldsymbol{y}}}(d,\,z),\,{M}_{{\boldsymbol{z}}}(d,\,z)]$ averaged over the locus of points (*d*, *z*)where *d* is the perpendicular distance from the domain wall of the OOMMF pattern and *z* is the depth within the sample: Additionally, *α*(*d*, *z*) is the angle between the in-plane component and the adjacent domain wall. When *α*(*d*, *z*) = 0 or π, the in-plane magnetization is parallel to the domain wall indicating a Bloch domain wall, whereas when *α*(*d*, *z*) = *π*/2, we have a Neel wall. This angle is allowed to vary as a function of *d* and *z* to allow for a smooth Neel/Bloch transition to be included in the model. The procedure of extracting the average values of $[{M}_{{\boldsymbol{x}}}(d,\,z),\,{M}_{{\boldsymbol{y}}}(d,\,z),\,{M}_{{\boldsymbol{z}}}(d,\,z)]$ and *α*(*d*, *z*)from an OOMMF simulation output is explained graphically in Fig. 1 for the case of the locus of points 15 nm from the network of domain walls.

The interaction between the X-rays and the sample is governed by the Eq. (2)^39^.

2 $${f}^{n}=({{\boldsymbol{e}}}_{n}\cdot {{\boldsymbol{e}}{\boldsymbol{^{\prime} }}}_{n}){f}_{c}^{n}+i({{\boldsymbol{e}}}_{n}\times {{\boldsymbol{e}}{\boldsymbol{^{\prime} }}}_{n})\cdot {{\boldsymbol{M}}}^{n}{f}_{m1}^{n}+({{\boldsymbol{e}}}_{n}\cdot {{\boldsymbol{M}}}^{n})({{\boldsymbol{e}}{\boldsymbol{^{\prime} }}}_{n}\cdot {{\boldsymbol{M}}}^{n}){f}_{m2}^{n},$$

where ${f}_{c}^{n}$, ${f}_{m1}^{n}$, and ${f}_{m2}^{n}$, are the X-ray form factors for the charge scattering, magnetic dipole scattering and quadrupole scattering, respectively, and ${{\boldsymbol{e}}}_{n}$ and ${{\boldsymbol{e}}{\boldsymbol{^{\prime} }}}_{n}$ are the incoming and outgoing electric field vectors, respectively. For the 3d metals being studied in this case, only ${f}_{m1}^{n}$ has an appreciable influence, and the consequence of this equation is that the magnetic contrast is proportional to the dot product between the Poynting vector of the incoming radiation and magnetization vector at each point in the sample. Near to an absorption edge, ${f}_{m1}^{n}$ exhibits a strong peak, meaning that the experiment is tuned to resonance near the Cobalt L_3_ edge to probe the magnetic structure within the Co/Pd layer and near to the Nickel L_3_ edge for the NiFe layer.

To simulate the diffraction pattern, for optically thin samples and low incidence angles with respect to the film normal, one may simply use the projection approximation to calculate the film transmission and then perform free-space propagation to arrive at the expected diffraction pattern. Near to the absorption edge, however, we have a strong interaction between the radiation and the sample, making necessary the use of slower multiple slice propagation as with our previous work^11^ – especially at higher incidence angles. This multiple slice propagation corrects for the Ewald Sphere curvature, and also the asymmetry observed in the diffracted intensity, where greater absorption of X-rays with a longer optical path within the sample results in an increasing diffracted intensity asymmetry as the incidence angle increases.

Full experimental details are presented at the end of the article, however, a short summary is stated here: The multilayer samples are placed in a soft X-ray beam tuned to the L3 resonances of either Ni or Co and rotated about the stripe domain axis, the resulting scattering signal being due to the magnetic contrast from the component of sample magnetisation parallel to the incident beam. Measurements are made at angles from normal incidence through to 74 degrees from normal, using progressively longer exposure times to compensate for sample absorption.

## Results and Discussion

This section will be divided in two: firstly, the experimental results for each sample are presented alongside the best fitting theoretical model, and subsequently, we assess the confidence in our fits by examining the sensitivity of the theoretical model with respect to changes in the anisotropy parameter *K*.

**Figure 2:**
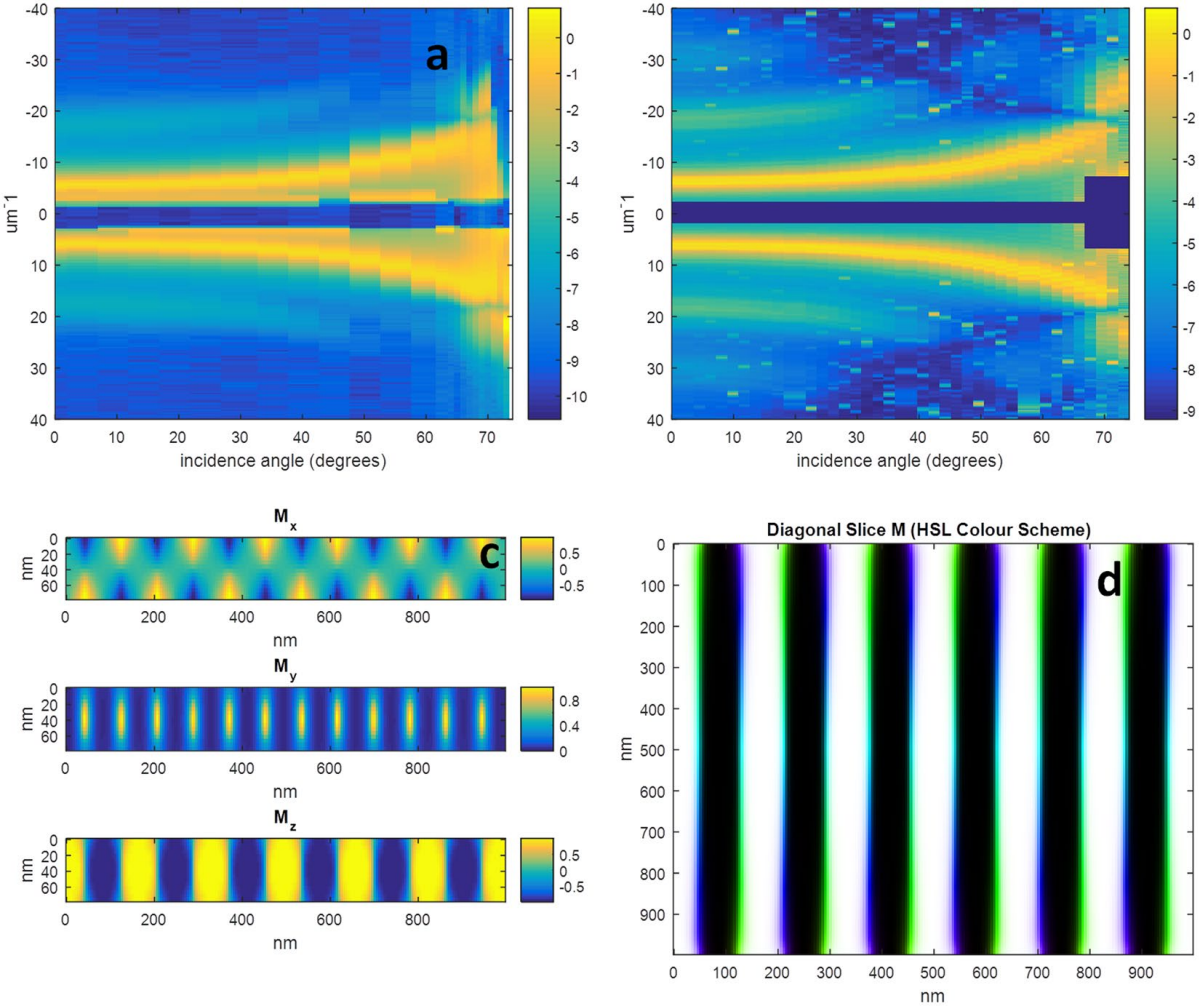
Results for the NiFe0 sample. (**a**) The experimental results showing the natural logarithm of the diffracted intensity versus incidence angle and scattering vector. (**b**) The equivalent theoretical results, (**c**) cross section of the micromagnetic simulation used to generate (**b**,**d**) an HSL colour image of the simulated magnetization vector field sampled along a diagonal slice, showing the transition from Neel to Bloch and back to Neel domain walls. Each vertical line in (**a**,**b**) is a normalized lineout plot of the diffraction pattern resulting from the corresponding incidence angle as described in the text, and the colour bar represents the natural logarithm of this normalized diffracted intensity. The colour bars in (**c**) represent the magnetization normalized between −1 and 1, and in (**d**) the black/white colour scale represents the out of plane component, and the colour the orientation of the transverse component from −pi to pi. The dark wide horizontal lines through the centre of (**a**,**b**) are due to the beam stop.

The control sample consisting of a [Co(0.8 nm)/Pd(0.8 nm)] × 50 multilayer (referred to hereforth as the “NiFe0” sample), corresponding to the Co/Pd multilayer system without NiFe layers, and the results and simulations for this sample are shown in Fig. 2. The figure compares experimental results with the best fitting theoretical model – generated with parameters *A*, *K* and M_s_ equal to 1.6 × 10^−11^ Jm^−1^, 2.5 × 10^5^ Jm^−3^, and 6.8 × 10^5^ Am^−1^, respectively.

We observed a stripe domain period of 160 nm and a primarily out-of-plane domain pattern with small closure domains consistent with previous observations^11^. The theoretical model allows for the inclusion of both Bloch and Neel domain walls, however, whilst rotating about the stripe domain axis, only the Neel walls are visible to the X-rays according to Eq. (2). Some measurements were performed rotating about the axis perpendicular to the stripes, however, it was not possible to separate the small Bloch walls from the dominant out-of-plane component of the scattering signal. As is the case with stripe domain samples, all interesting data is perpendicular to the stripe axis in reciprocal space, allowing us to present the data as lineout plots, where each lineout represents the diffracted intensity integrated along the stripe axis. The complete dataset for each sample is thus presented as a collection of lineouts, with each line normalized against its maximum intensity to allow the straightforward comparison between the measured diffraction at distinct incidence angles. The diffraction pattern for all samples displays the behaviour where the 1^st^ order diffraction peaks shift to higher *q* according to 1/*cos*(*θ*), where *θ* is the incidence angle. However, there are angles *θ* where the intensity suffers a sharp diminution - referred to as *critical angles* in our previous work^11^. These occur when the spatial magnetic contrast reduces to zero from the point of view of the incident beam, and the angle *θ* at which they occur is what provides information regarding the magnetic morphology. As will be fully explained in the subsequent section, small changes of the magnetic anisotropy parameter *K* as inputted into the micromagnetic simulations are shown to shift the *critical angles* to the order of a few degrees.

**Figure 3:**
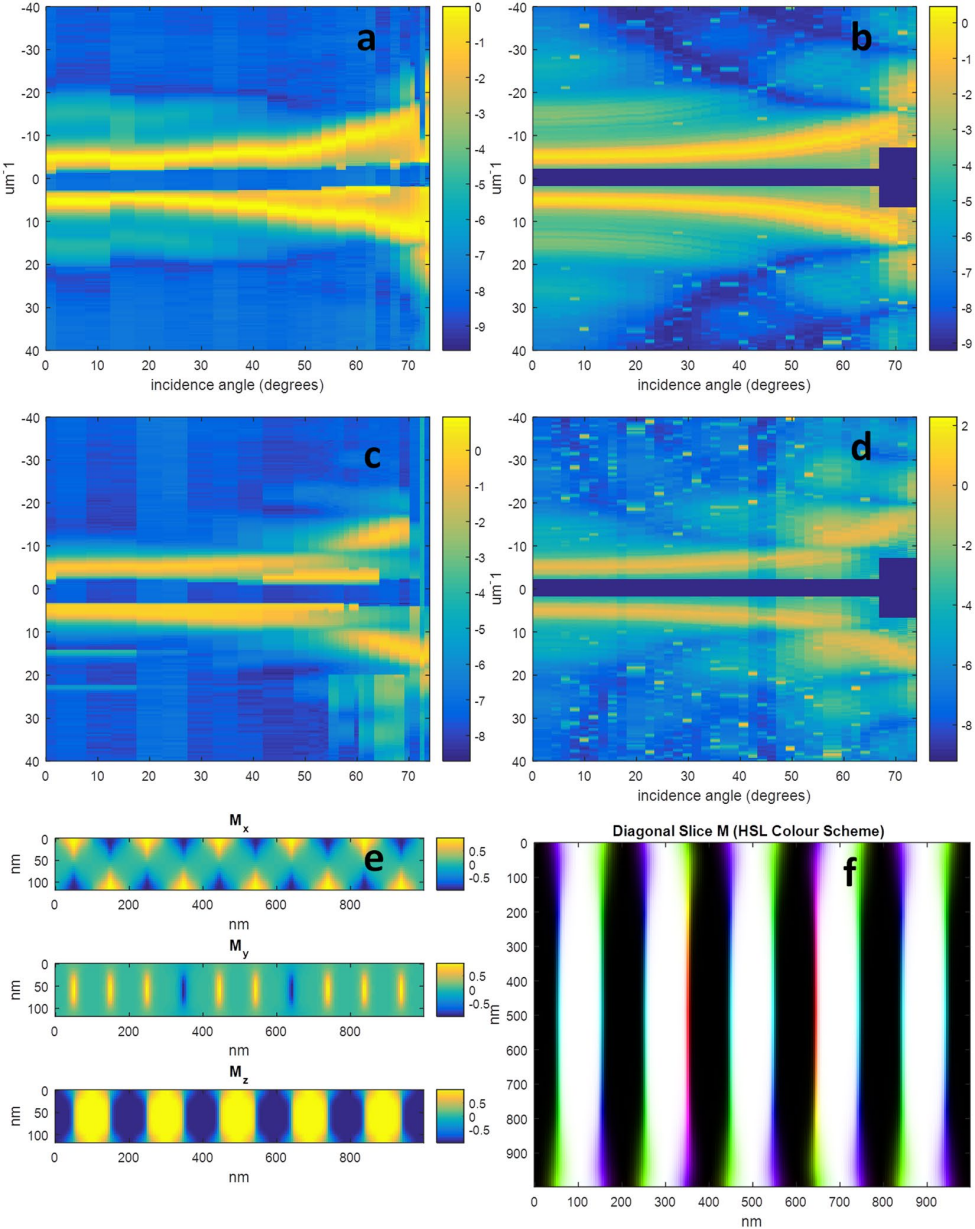
Results for the NiFe20 sample. (**a**) The experimental results at 776 eV showing the natural logarithm of the diffracted intensity versus incidence angle and scattering vector. (**b**) The equivalent theoretical results, (**c**) the experimental results at 851 eV, (**d**) the equivalent theoretical results, (**e**) cross section of the micromagnetic simulation used to generate (**b**,**d**,**f**) an HSL colour image of the simulated magnetization vector field sampled along a diagonal slice, showing the transition from Neel to Bloch and back to Neel domain walls. Each vertical line in (**a**–**d**) is a normalized lineout plot of the diffraction pattern resulting from the corresponding incidence angle as described in the text, and the colour bar represents the natural logarithm of this normalized diffracted intensity. The colour bars in (**e**) represent the magnetization normalized between −1 and 1, and in (**f**) the black/white colour scale represents the out of plane component, and the colour the orientation of the transverse component from −pi to pi. The dark wide horizontal lines through the centre of (**a**–**d**) are due to the beam stop.

The second sample whose results are shown in Fig. 3 consists of the NiFe(20 nm)/Pd(0.8 nm)/[Co(0.8 nm)/Pd(0.8 nm)] × 50/NiFe(20 nm) multilayer, and is referred to as the “NiFe20” sample from this point onwards. This sample can be thought of as a “sandwich” consisting of two 20 nm NiFe bread slices and a 50 repeat Co/Pd multilayer as filling. In this case we measured at both 776 and 851 eV for sensitivity to the cobalt and nickel, respectively. A stripe domain period of 200 nm was observed, however, for all measurements with an incidence angle greater than 15 degrees from normal incidence a small misalignment of the sample was detected. This misalignment led to diffraction peaks being observed at smaller scattering angles compared to if the sample were perfectly aligned. This misalignment occurred after a remounting of the sample and has been accounted for in the theoretical modelling by rotating the stripe axis by 10 degrees with respect to the rotation axis. Regarding higher order diffraction peaks, the only ones which were reliably observed for this sample were those seen at 776 eV between normal incidence and 45 degrees from the normal. For the 851 eV measurements, the signal which appears to be higher angle scatter is in fact believed to be charge scattering background due to its differing morphology compared with theoretical plots, its presence even when the lineout was calculated in regions where the magnetic scattering was not expected to be present, and its observed evolution into a typical diffuse SAXS ring at normal incidence corresponding to a disordered matrix of 8 nm nanocrystals. To reliably observe the weak higher order diffraction in this case, illumination with circularly polarized coherent light along with a reduced beam size will be necessary to allow the post-experiment separation of the magnetic and charge components. Due to this limitation, the match between theory and experiment was based only upon the first order scatter, and suggests that the NiFe layers exhibit in-plane and out-of-plane magnetisation in approximately equal quantities. The simulation parameters for this sample were *A*, *K* and M_s_ equal to 1.3 × 10^−11^ Jm^−1^, 3.0 × 10^5^ Jm^−3^, and 6.8 × 10^5^ Am^−1^, respectively for the CoPd layer, and *A*, *K* and M_s_ equal to 1.3 × 10^−11^ Jm^−1^, 1.0 × 10^5^ Jm^−3^, and 5.4 × 10^5^ Am^−1^, respectively for the NiFe layers. The weighted average of the anisotropy energy is equal to the value of 6.5 × 10^5^ Am^−1^, extracted from the measured saturation field value of 7.2 kOe.

**Figure 4:**
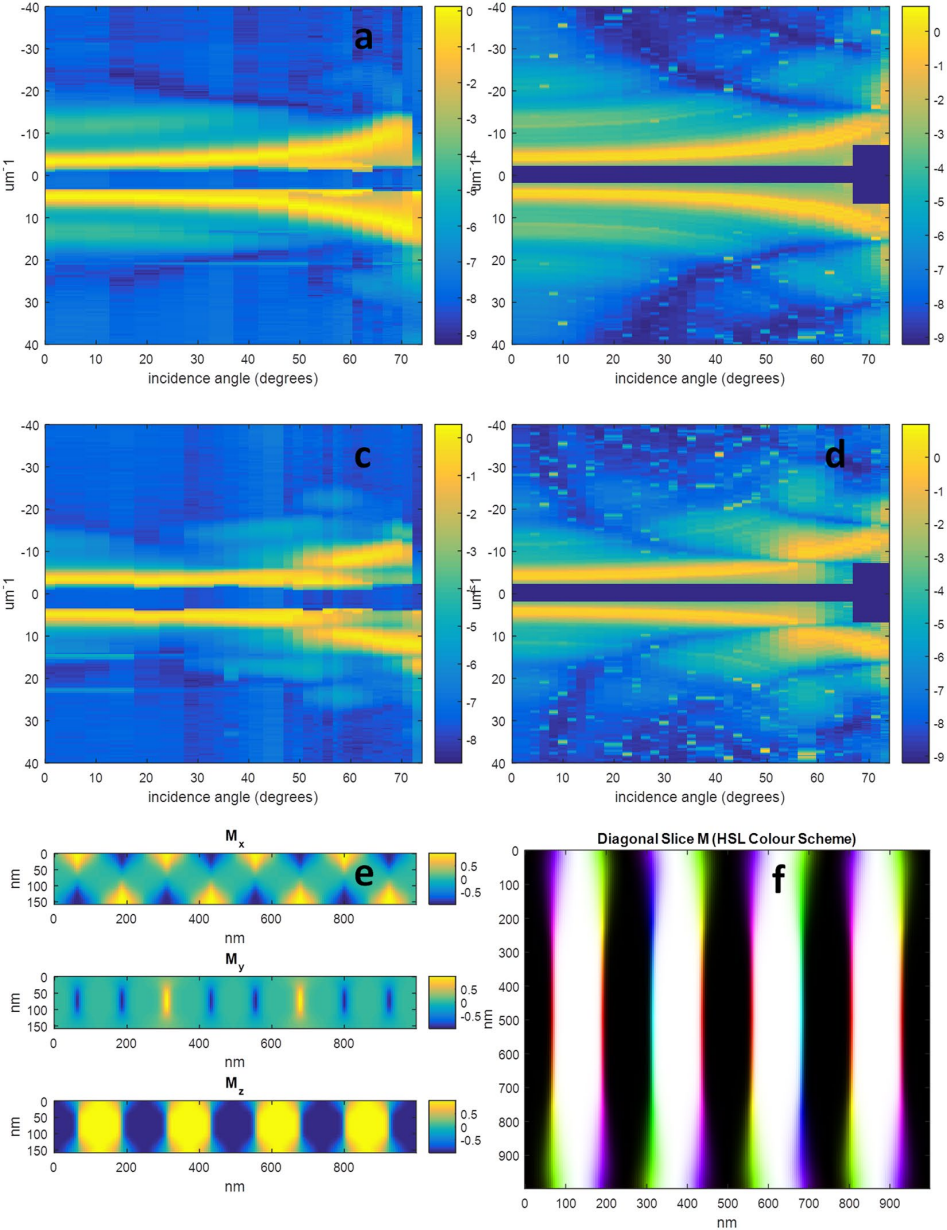
Results for NiFe40 sample. (**a**) The experimental results at 776 eV showing the natural logarithm of the diffracted intensity versus incidence angle and scattering vector. (**b**) The equivalent theoretical results, (**c**) the experimental results at 851 eV, (**d**) the equivalent theoretical results, (**e**) cross section of the micromagnetic simulation used to generate (**b**,**d**,**f**) an HSL colour image of the simulated magnetization vector field sampled along a diagonal slice, showing the transition from Neel to Bloch and back to Neel domain walls. Each vertical line in (**a**–**d**) is a normalized lineout plot of the diffraction pattern resulting from the corresponding incidence angle as described in the text, and the colour bar represents the natural logarithm of this normalized diffracted intensity. The colour bars in (**e**) represent the magnetization normalized between −1 and 1, and in (**f**) the black/white colour scale represents the out of plane component, and the colour the orientation of the transverse component from −pi to pi. The dark wide horizontal lines through the centre of (**a**–**d**) are due to the beam stop.

The third sample whose results are shown in Fig. 4 consists of the a NiFe(40 nm)/Pd(0.8 nm)/[Co(0.8 nm)/Pd(0.8 nm)] × 50/NiFe(40 nm) multilayer, and is referred to as the “NiFe40” sample from this point onwards. Our measurements are consistent with the closure domains being located almost entirely in the NiFe layers as is inferred by the strong 3^rd^ order diffraction being present at normal incidence in the measurements at the cobalt edge, and also to a weaker extent in the measurements at the nickel edge – the latter suggesting a degree of sharpness in the change between parallel and anti-parallel magnetisation in the NiFe layers. The above implies additionally that the domain walls in the Co/Pd layer are almost entirely of the Bloch type. In this case, it was possible to utilize the position of the *critical angles* present in the higher order scatter in the adjustment of the anisotropy parameter *K* to best fit the data; something not possible with the NiFe20 sample. This allows a greater degree of confidence in concluding the morphology of the sample proposed in Fig. 4e,f. Despite this greater confidence compared with the previous samples, coherent illumination could even further improve the reliability of the analysis. The simulation parameters for this sample were *A*, *K* and M_s_ equal to 1.9 × 10^−11^ Jm^−1^, 3.4 × 10^5^ Jm^−3^, and 6.8 × 10^5^ Am^−1^, respectively for the CoPd layer, and *A*, *K* and M_s_ equal to 1.9 × 10^−11^ Jm^−1^, 1.0 × 10^5^ Jm^−3^, and 5.4 × 10^5^ Am^−1^, respectively for the NiFe layers.

## Evaluation of the Measurement Sensitivity

As discussed in the section on the micromagnetic simulations, there is a degree of uncertainty regarding the correct anisotropy parameters to use for modelling the magnetisation within the NiFe layers. As other authors have done, for example Tryputen *et al*.^40^, constant micromagnetic parameters are used for each species throughout the process, and in our case setting the anisotropy in the NiFe layers to zero in the micromagnetic simulations predicted *some* but not all of the induced out-of-plane magnetisation observed in the NiFe layers. Letting the value of *K* take on a positive (out-of-plane) value allowed a fairly good fit to be obtained as is demonstrated in Figs 3 and 4. To further investigate, we set out to evaluate the sensitivity of the X-ray scattering simulations for each of the samples to changes in the magnetic morphology due to adjustments in the anisotropy parameter.

**Figure 5:**
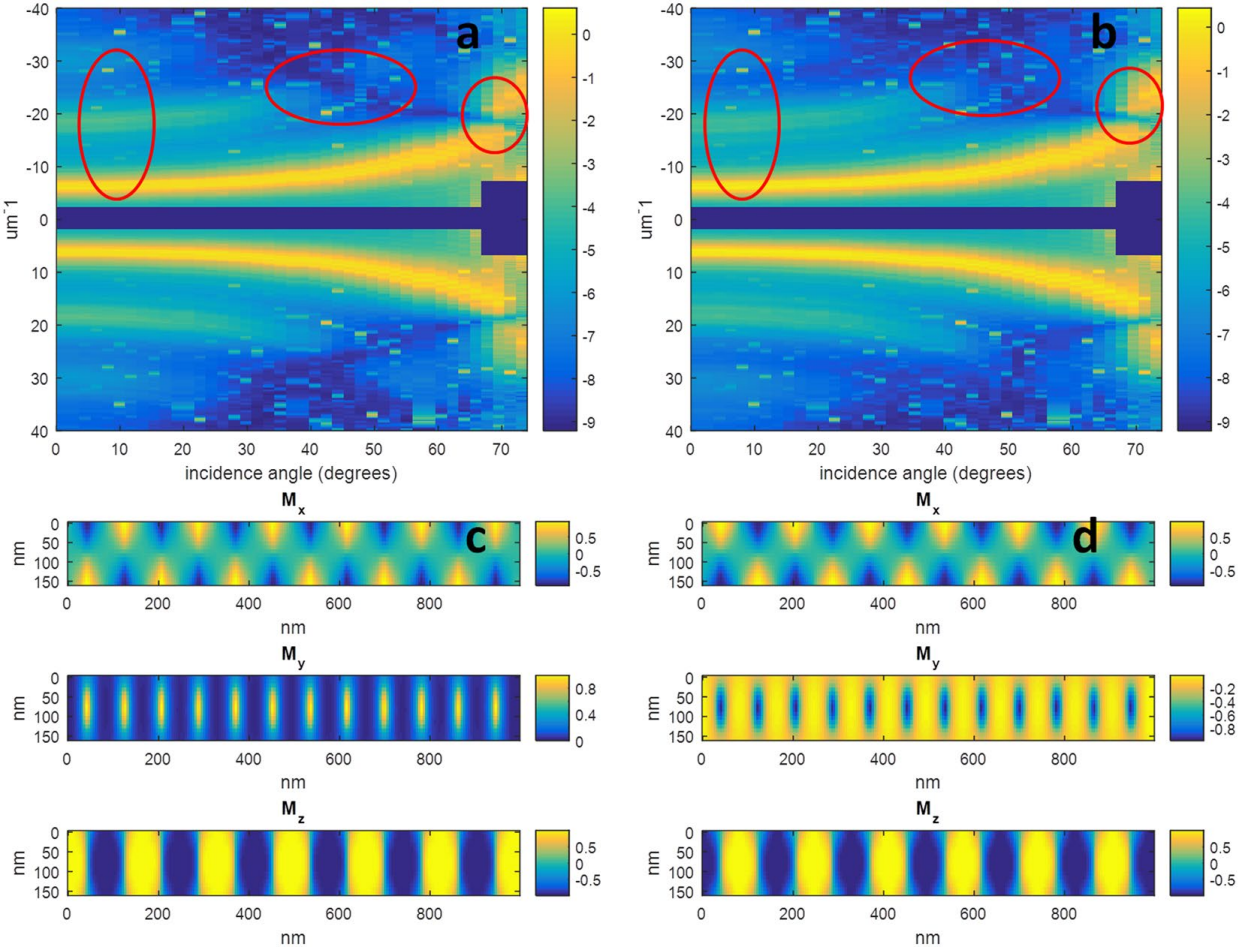
(**a**) Natural logarithm of the simulated of the X-ray scattering intensity predicted for the NiFe0 sample using the same parameters as in Fig. 2 with *K* = 2.5 × 10^5^ Jm^−3^ in comparison with scattering generated using a value of b) *K* = 1.5 × 10^5^ Jm^−3^. The ellipses on (**a**,**b**) help to guide the reader to the chief differences between the two figures: At normal incidence, the higher order diffraction increases with increasing magnetic anisotropy, and the critical angles decrease with increasing anisotropy. In this particular case, the shift in the critical angle of the 3^rd^ order diffraction is notably more important than the case of the 1^st^ order diffraction which is of the order of only 1 degree. (**c**) Cross section through the micromagnetic simulation output for *K* = 2.5 × 10^5^ Jm^−3^ (identical to Fig. 2), and (**d**) the cross section through the micromagnetic simulation output for *K* = 1.5 × 10^5^ Jm^−3^.

In Fig. 5, we demonstrate the effect of changing the anisotropy of the control sample by showing the difference between the simulation for the NiFe0 sample calculated for an anisotropy energy of 1.5 × 10^5^ Jm^−3^, and the simulation used in Fig. 2 with an anisotropy energy of 2.5 × 10^5^ Jm^−3^. In this particular case, the *critical angle* of the 1^st^ order diffraction peak shifted by approximately 1 degree, however, the behavior of the 3^rd^ order peak at lower incidence angles suffered more important changes: In the case of lower anisotropy it is substantially weaker, but is present to higher incidence angles than in the case of the higher anisotropy simulation. This observation further demonstrates the need for a good quality incident radiation intensity measurement in future experiments to allow quantitative comparisons between the diffraction intensity at different incident angles – an important limitation with the experiment reported in this manuscript.

**Figure 6:**
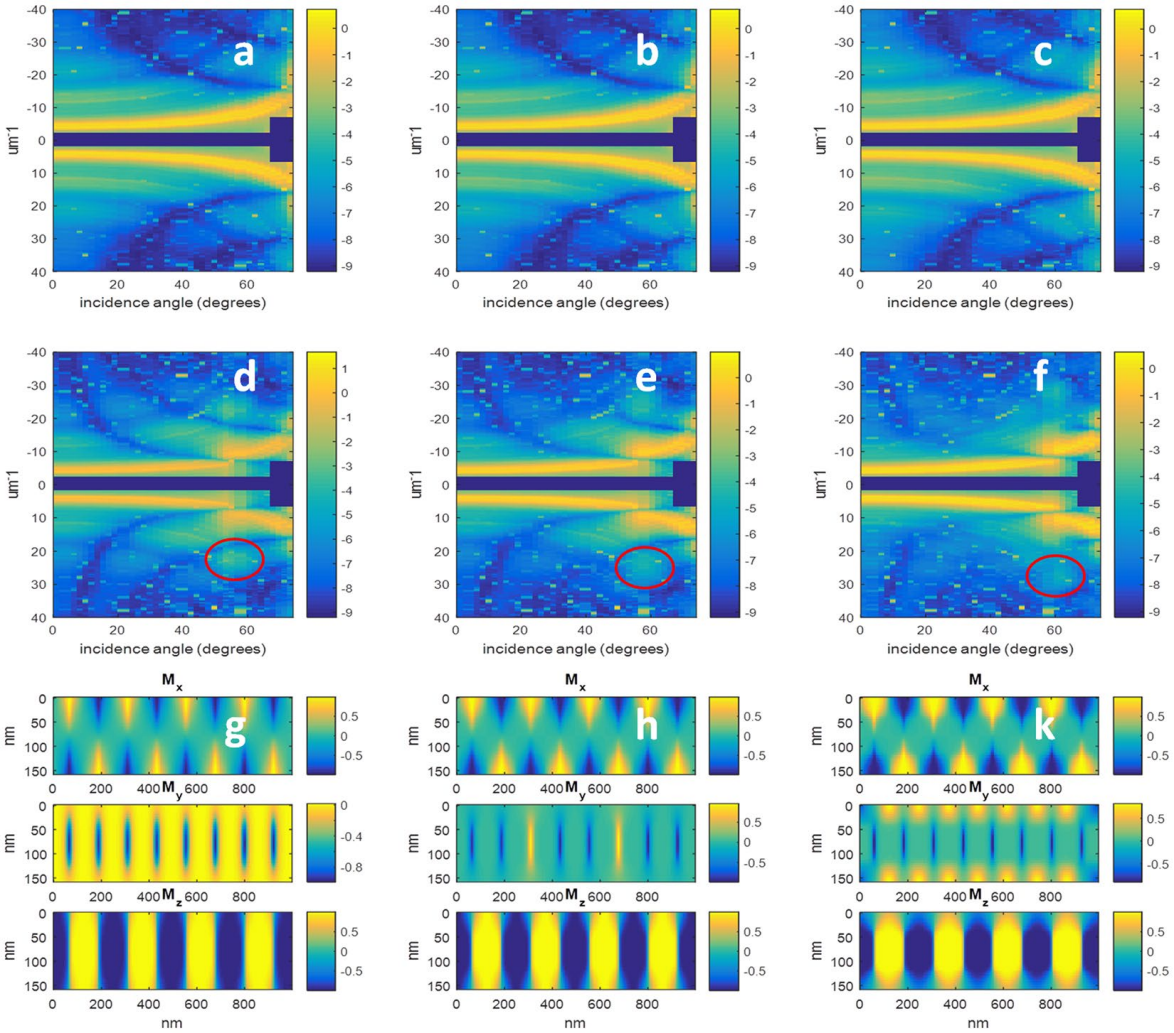
Natural logarithm of the simulated of the X-ray scattering intensities predicted for the NiFe40 sample with different simulation conditions. (**a**–**c**) And the cobalt edge simulated scattering signals for the samples with NiFe anisotropy parameter *K* = 2.0, 1.0 and 0 × 10^5^ Jm^−3^, respectively, (**d**–**f**) are the nickel edge simulated scattering signals for the samples with NiFe anisotropy parameter *K* = 2.0, 1.0 and 0 × 10^5^ Jm^−3^, respectively, and (**g**–**k**) are cross sections through the micromagnetic simulation output for each of the above respective cases. Here the middle 80 nm represents the CoPd multilayer stack, and the top and bottom 40 nm represent the NiFe layers. In all of the above simulations, the value of *K* for the CoPd layer was set so as to maintain an average value of *K* = 2.21 × 10^5^ Jm^−3^ for each case. The red oval in (**d**–**f**) marks a lobe in the 3^rd^ order diffraction whose position is sensitive to the induced out of plane anisotropy in the NiFe layers.

As discussed in the section on the micromagnetic simulations, there was a degree of uncertainty regarding the ideal way to model the degree to which perpendicular anisotropy was induced into the NiFe layers. For comparison purposes, in Fig. 6 we show the predicted X-ray scattering from simulated versions of the NiFe40 sample using three different combinations of anisotropy parameters (and the average in each case conforming to the measured value of 2.21 × 10^5^ Jm^−3^). The first simulation assumes that the entirety of the sample has uniform saturation field *Hs*, providing values of *K* of 2.4 × 10^5^ Jm^−3^ and 2.0 × 10^5^ Jm^−3^ for the Co/Pd and NiFe layers respectively. The second simulation is a repeat of that of Fig. 4 with *K* = 3.4 × 10^5^ Jm^−3^and 1.0 × 10^5^ Jm^−3^, and the third assumes totally isotropic NiFe layers and a value of *K* = 4.4 × 10^5^ Jm^−3^ in the CoPd layer. In each of the three cases, the scattering pattern at the cobalt edge is practically identical, demonstrating that the closure domains in all cases are located in almost in their entirety within the NiFe layers. This cannot be said, however, for the predicted scattering at the nickel edge, where there are important differences between the different results shown in Fig. 6d,e,f. The first observation is that the 3^rd^ order scatter at normal incidence ranges from being strongly present in 6d to barely visible in the case of 6 f. A second observation is the shift of the critical angle of the 1^st^ order diffraction from an angle in the low 50’s in the case of 6d to around 60 degrees in the case of 6 f. Associated with this observation, the lobe in the 3^rd^ order diffraction marked by the red oval in Fig. 6d–f moves in position from incidence angles in the mid 50’s to the low 60’s between 6d and 6e. In Fig. 6g–k, there is a marked intrusion of the out-of-plane domain into the superficial NiFe layers, however, the size of the intrusion decreases from 6 g through to 6k. In 6 g and 6 h there is also a partial intrusion of the Bloch/Neel transition zone into the superficial NiFe layers. Given an estimated uncertainty of ±1° in the orientation of the sample in the scattering chamber, and the differences in angular position of the features listed above between the figures being greater than this amount, we tentatively conclude that our experimental results suggest an induced out of plane magnetization in the NiFe layers greater than that predicted by micromagnetic simulations obtained assuming *K* = 0 in the NiFe layer. This observation could possibly be further tested in a future experiment by inserting a thin layer of a different material at different depths within the sample to be magnetised by the proximity effect, and subjecting this new sample to X-ray scattering measurements in both reflection and transmission geometry to probe the 2D magnetisation profile of this thin inserted layer.

The qualitative behaviour of the simulations related to the NiFe20 sample was similar to that discussed here for the NiFe40 sample, however, observed differences were reduced due to the smaller amount of NiFe present.

## Future Perspectives

This work was performed on moderately disordered stripe domain samples, which allowed for the straightforward collection of diffraction data based upon rotation about only one rotation axis. For more complicated magnetic nanostructures, two axes of rotation will be required, along with further a-priori information about the magnetic morphology – at least in two dimensions in order to seed the scattering simulations. As with any scattering technique, the benefits of higher resolution and a simpler experimental setup come at the cost of the fact that the precision of the results obtained will always be inferior to those obtained using a direct imaging method such as scanning microscopy. This is in a manner analogous the study of crystals using X-ray diffraction – there becomes a point in terms of complexity where single crystal diffraction is vastly superior to powder diffraction in terms achieving a structure solution. One manner to study more complex samples could involve the insertion of a single thin layer of another metal to be magnetized by the proximity effect, and to perform the scattering measurements either in reflection or transmission geometry at the absorption edge of this inserted metal. Such a method could potentially reduce the problem from 3 to 2 dimensions allowing conclusions to be extracted with greater precision regarding the magnetisation profile within thicker samples. For labyrinth domain samples, our previous work^11^ suggested that the technique presented here would be useful for the characterisation of their domain morphology, and more precise work is underway to confirm this. The precision and resolution will however not be to the same level as that demonstrated here, due to the fact that the presence of stripes allows for  an amplification of the diffraction intensity in a similar manner to that observed with Bragg diffraction.

## Conclusions

Compared with our previous work^11^, this paper represents an advance both in the area of the interpretation and modelling of the system, and the use of the transmission mode X-ray resonant magnetic scattering technique for examining the boundary effects at a buried interface between the Co/Pd multilayers and the NiFe layers. In terms of modelling, one of the weaknesses of our previous work^11^ was the inability to explain the shift in the *critical angles*, which the more realistic modelling employed here, has enabled us to use this observation as a probe of the magnetic anisotropy. For both the NiFe20 and NiFe40 samples, the observed out-of-plane magnetisation was tentatively found to be greater than that which would be expected from micromagnetic simulations performed assuming the NiFe layer to be purely isotropic. Based on this observation, further work is needed in order to quantify the magnitude of the observed out-of-plane magnetisation in the NiFe layers.

The recipe developed here to insert micromagnetic simulations directly into the scattering simulation code will allow us in the near future to apply this technique to a variety of sample classes, especially in cases where the sample is subject to different external fields, temperatures or in time resolved measurements at a free electron laser where the application of scanning microscopy techniques such as those employed by Donnelly *et al*.^29,30^ would not be practical.

The issue of flux is also key because of its direct relation with the resolution: Around the time of the submission of this manuscript the authors attempted to measure the NiFe40 sample studied in this work and some similar samples with ptychography at the Advanced Light Source^41^ but failed to detect sufficient usable scatter to a resolution beyond the 45 nm half period provided by the zone plate. The advent of 4^th^ generation machines in the coming years could help in this regard, and the full analysis of this experiment and a discussion of intrinsic resolution limitations will be subject to an upcoming manuscript.

It remains a moot point whether the use of reflection geometry or transmission geometry XRMS is advantageous for the study of samples such as those in this current manuscript, especially for multilayer samples where the large quantity of interfaces allows for depth sensitive measurements in both modalities. For single layer samples, such as the NiFe studied here, reflection geometry is limited in sensitivity due to the lack of inter-material interfaces where reflection may occur. By calculating the reflection and transmission coefficients according to references^42–45^, the same simulation procedure employed in this work may be generalized for reflection geometry, and we are currently working on a theoretical study to examine under which conditions XRMS is more sensitive in either reflection or transmission geometry.

Finally, the imprinting of the PMA multilayer domains in the soft magnetic material with in-plane anisotropy, confirmed in this work and corroborating the paper of Yurui *et al*.^10^, has potential applications in tuning magnetic properties such as FMR as demonstrated by Yurui *et al*.^10^. In this sense, this work is an important step towards providing a valuable experimental tool for investigations in magnetic buried interfaces.

## Supporting Data

The datasets generated during and/or analyzed during the current study are available from the corresponding author on reasonable request.

## Methods - Sample Fabrication and Experimental Procedure

The multilayer samples were fabricated on 3 × 3 mm^2^ silicon nitride membranes of 50 nm thickness at the Magnetism Laboratory of the Universidad de Santiago de Chile by an INTERCOVAMEX magnetron sputtering system. The base pressure in the chamber was less than 8 × 10^−7^ Torr, and the working pressure of argon was 3 mTorr. The multilayer samples consist of [Co(0.8 nm)/Pd(0.8 nm)] × 50 (the “NiFe0” sample), NiFe(20 nm)/Pd(0.8 nm)/[Co(0.8 nm)/Pd(0.8 nm)] × 50/NiFe(20 nm) (the “NiFe20” sample), and NiFe(40 nm)/Pd(0.8 nm)/[Co(0.8 nm)/Pd(0.8 nm)] × 50/NiFe(40 nm) (the “NiFe40” sample). In every multilayer, a Pd (1 nm) seed layer was used, and on the surface of all the samples, a Pd (1 nm) layer was deposited to prevent oxidation. The samples were subsequently subject to an AC demagnetization procedure with around 50 sweeps of an external magnetic field in an exponentially decaying fashion from 1 T down to zero in order to align the domains in a (somewhat disordered) stripe pattern. The hysteresis curves of the samples were measured using a mini 5 Tesla VSM from Cryogenic Ltd., at a temperature of 290 K. For the VSM measurements, the films where cut in small 5 × 5 mm squares and measured with field applied only in the plane of the films for case of the pure NiFe control films. For the Co/Pd films (NiFe0, NiFe20 and NiFe40) the measurements were made with the orientation of the applied field both parallel and perpendicular to the plane of the films. Full details of the VSM characterisation including the hysteresis loops can be found in the supplementary material file.

The measurements were carried out at the soft X-ray scattering endstation of the U11A-PGM beamline of the UVX accelerator of the Brazilian Synchrotron Light Laboratory (UVX-LNLS) in Campinas, Brasil^46^, at the energies of 776 eV for probing the cobalt layer and at 851 eV for probing the NiFe layer. The samples were mounted with the stripes parallel to the rotation axis, and measurements were taken at a range of angles from normal incidence up to 75 degrees from the normal. Near to normal incidence, the observed qualitative properties of the diffraction were observed to vary only slowly with the increment of the incidence angle allowing measurements to be made at an interval of 5 degrees. At higher incidence angles however, measurements were taken every 1 or 2 degrees. Exposure time varied between a total of 0.1 seconds at normal incidence to several minutes at the highest incidence angles.

Certain experimental parameters such as the horizontal beam size (near to 1 mm) and coherence length (around 10 μm) were sub-optimal in the current experiment owing to the fact that the now decomissioned UVX-LNLS was one of the few remaining second generation machines still in operation and will soon be replaced by the fourth generation SIRIUS-LNLS machine. As such, we were unable to perform the usual procedure of calculating the difference of both clockwise and anticlockwise circular helicities to separate the magnetic from its non-magnetic contribution – a factor which limited our resolution for some samples. We thus expect that newer measurements will exhibit a higher level of sensitivity compared with the results presented here.

## Supplementary information

VSM Measurement Details and Hysteresis Loops

## References

[1] Schafer R, Hubert A (1998). Domains in soft magnetic materials. J Phys Iv.

[2] Durr HA (1999). Chiral magnetic domain structures in ultrathin FePd films. Science.

[3] Ryu K-S, Thomas L, Yang S-H, Parkin SSP (2012). Current Induced Tilting of Domain Walls in High Velocity Motion along Perpendicularly Magnetized Micron-Sized Co/Ni/Co Racetracks. Appl Phys Express.

[4] Schuller IK, Kim S, Leighton C (1999). Magnetic superlattices and multilayers. J Magn Magn Mater.

[5] Allwood DA (2005). Magnetic domain-wall logic. Science.

[6] Li D (2018). Current-Induced Domain Wall Motion and Tilting in Perpendicularly Magnetized Racetracks. Nanoscale research letters.

[7] Fert A, Cros V, Sampaio J (2013). Skyrmions on the track. Nature nanotechnology.

[8] Zhang, X. C. *et al*. Skyrmion-skyrmion and skyrmion-edge repulsions in skyrmion-based racetrack memory. *Sci Rep-Uk* **5** (2015). 10.1038/srep07643 PMC4284505 25560935

[9] Krause S, Wiesendanger R (2016). SPINTRONICS Skyrmionics gets hot. Nat Mater.

[10] Yurui W (2019). Metastable magnetic bubble in [Co/Pd] 4 /Py multilayers. Journal of Physics D: Applied Physics.

[11] Flewett, S. *et al*. Three-dimensional characterization of Co/Pd multilayer thin films using resonant soft x-ray scattering. *Phys Rev B* **95** (2017).

[12] Bagschik, K. *et al*. Employing soft x-ray resonant magnetic scattering to study domain sizes and anisotropy in Co/Pd multilayers. *Phys Rev B* **94** (2016).

[13] Bagschik K (2016). Spatial coherence determination from the Fourier analysis of a resonant soft X-ray magnetic speckle pattern. Opt Express.

[14] Dudzik E (2000). Influence of perpendicular magnetic anisotropy on closure domains studied with x-ray resonant magnetic scattering. Phys Rev B.

[15] Miguel, J. *et al*. X-ray resonant magnetic scattering study of magnetic stripe domains in *a*-GdFe thin films. *Phys Rev B* **74** (2006).

[16] van der Laan G (2004). Magnetic anisotropy of aligned magnetic stripe domains in FePd studied by soft X-ray resonant magnetic scattering, magnetic force microscopy and micromagnetic modeling. Superlattice Microst.

[17] Kortright JB (2013). Resonant soft X-ray and extreme ultraviolet magnetic scattering in nanostructured magnetic materials: Fundamentals and directions. J Electron Spectrosc.

[18] Fink, J., Schierle, E., Weschke, E. & Geck, J. Resonant elastic soft x-ray scattering. *Rep Prog Phys* **76** (2013). 10.1088/0034-4885/76/5/056502 23563216

[19] Fin, S. *et al*. In-plane rotation of magnetic stripe domains in Fe_1-x_Ga_x_ thin films. *Phys Rev B* **92** (2015).

[20] Chauleau, J. Y. *et al*. Chirality in Magnetic Multilayers Probed by the Symmetry and the Amplitude of Dichroism in X-Ray Resonant Magnetic Scattering. *Phys Rev Lett* **1 20** (2018). 10.1103/PhysRevLett.120.037202 29400492

[21] Streubel, R. *et al*. Retrieving spin textures on curved magnetic thin films with full-field soft X-ray microscopies. *Nature Communications* **6** (2015). 10.1038/ncomms8612 PMC4506513 26139445

[22] Boulle O (2016). Room-temperature chiral magnetic skyrmions in ultrathin magnetic nanostructures. Nature nanotechnology.

[23] Blanco-Roldan, C. *et al*. Nanoscale imaging of buried topological defects with quantitative X-ray magnetic microscopy. *Nature Communications* **6** (2015). 10.1038/ncomms9196 PMC4569793 26337838

[24] Suzuki, M. *et al*. Three-dimensional visualization of magnetic domain structure with strong uniaxial anisotropy via scanning hard X-ray microtomography. *Appl Phys Express* **11** (2018).

[25] Hierro-Rodriguez A (2018). 3D reconstruction of magnetization from dichroic soft X-ray transmission tomography. J Synchrotron Radiat.

[26] Popescu, H. *et al*. Four-state magnetic configuration in a tri-layer asymmetric ring. *Appl Phys Lett* **107** (2015).

[27] Donnelly, C. *et al*. Element-Specific X-Ray Phase Tomography of 3D Structures at the Nanoscale. *Phys Rev Lett* **114** (2015). 10.1103/PhysRevLett.114.115501 25839287

[28] Donnelly, C. *et al*. High-resolution hard x-ray magnetic imaging with dichroic ptychography. *Phys Rev B* **94** (2016).

[29] Donnelly, C. *et al*. Tomographic reconstruction of a three-dimensional magnetization vector field. *New J Phys* **20** (2018).

[30] Donnelly C (2017). Three-dimensional magnetization structures revealed with X-ray vector nanotomography. Nature.

[31] Lemesh, I., Buttner, F. & Beach, G. S. D. Accurate model of the stripe domain phase of perpendicularly magnetized multilayers. *Phys Rev B* **95** (2017).

[32] Kim H, You CY (2016). Embedded Object-Oriented Micromagnetic Frame (OOMMF) for More Flexible Micromagnetic Simulations. J Magn.

[33] Hashimoto S, Ochiai Y (1990). Co/Pt and Co/Pd multilayers as magneto-optical recording materials. J Magn Magn Mater.

[34] Shaw JM (2008). Reversal mechanisms in perpendicularly magnetized nanostructures. Phys Rev B.

[35] Kamberský V (1996). Domain wall theory and exchange stiffness in Co/Pd multilayers. J Magn Magn Mater.

[36] Lau JW, Liu X, Boling RC, Shaw JM (2011). Decoupling nucleation and domain-wall propagation regimes in (Co/Pd)_n_multilayer nanostructures. Phys Rev B.

[37] Liu Z (2011). Thickness dependent magnetization dynamics of perpendicular anisotropy Co/Pd multilayer films. J Magn Magn Mater.

[38] Gupta R, Gupta M, Gutberlet T (2008). Magnetization in permalloy thin films. Pramana.

[39] Hannon JP, Trammell GT, Blume M, Gibbs D (1988). X-Ray Resonance Exchange Scattering. Phys Rev Lett.

[40] Tryputen, L. *et al*. Magnetic structure and anisotropy of [Co/Pd]_5_/NiFe multilayers. *Phys Rev B* **91**, 014407, (2015).

[41] Yu Y-S (2018). Nanoscale Visualization of Magnetic Contrasts with Soft X-ray Spectro-Ptychography at the Advanced Light Source. Microsc Microanal.

[42] Zak J, Moog ER, Liu C, Bader SD (1990). Fundamental Magnetooptics. J Appl Phys.

[43] Zak J, Moog ER, Liu C, Bader SD (1990). Universal Approach to Magnetooptics. J Magn Magn Mater.

[44] Zak J, Moog ER, Liu C, Bader SD (1991). Magnetooptics of Multilayers with Arbitrary Magnetization Directions. Phys Rev B.

[45] Qiu ZQ, Bader SD (2000). Surface magneto-optic Kerr effect. Rev Sci Instrum.

[46] Cezar JC (2013). The U11 PGM beam line at the Brazilian National Synchrotron Light Laboratory. Journal of Physics: Conference Series.

