# Supplementary material for: Soft X-ray magnetic scattering studies of 3D magnetic morphology along buried interfaces in NiFe/CoPd/NiFe nanostructures
Source: Sci Rep. 2019 Oct 15;9:14823. doi: 10.1038/s41598-019-51098-9 (PMC6794309; doi:10.1038/s41598-019-51098-9)
Supplement: Supplementary file 1 — VSM Measurement Details and Hysteresis Loops [file 41598_2019_51098_MOESM1_ESM.pdf]

Soft X-ray magnetic scattering studies of 3D magnetic morphology along buried interfaces in NiFe/CoPd/NiFe nanostructures

Samuel Flewett, Thiago J. A. Mori, Alexandra Ovalle, Simón Oyarzún, Antonio Ibáñez, Sebastián Michea, Juan Escrig, Juliano Denardin

Supplementary Material: VSM Measurement Details and Hysteresis Loops

The hysteresis curves of the samples were measured using a mini 5Tesla VSM from Cryogenic Ltd., at a temperature of 290K. The VSM was previously calibrated with a standard YIG sphere.

The films where cut in small 5 x 5 mm squares and measured with field applied only in the plane of the films in case of the pure NiFe control films. For the Co/Pd films (NiFe0, NiFe20 and NiFe40) the measurements were made with the orientation of the field both parallel and perpendicular to the plane of the films.

The main uncertainly in the measurements of *Ms* comes from estimation of sample volumes, in the VSM calibration and in small differences in sample centering position. And finally, the noise level of the VSM in the saturation region (see Fig. S5) makes it impossible to distinguish between the final values of 6.2 x 10^5 Am^{-1} and 6.7 x 10^5 Am^{-1} for the saturation magnetizations of NiFe20 and NiFe40. (See Fig. S5). We estimate the total uncertainty on *Ms* and *Hs* to be ±10%, producing an uncertainty of ±14% when combined to estimate the anisotropy energy density *K*, using the expression $K = \frac{1}{2} H_s M_s$.

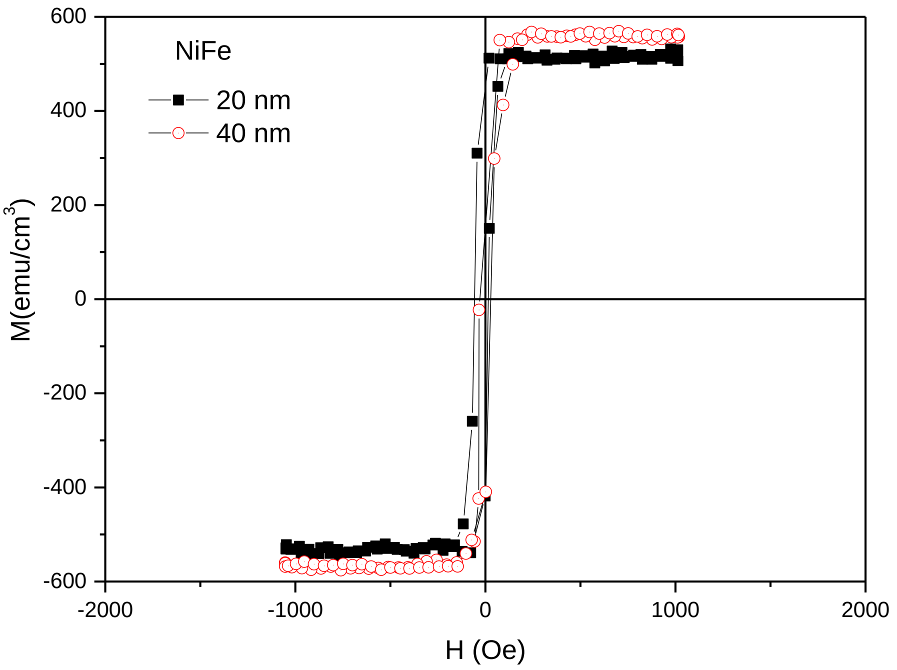

Supplementary Fig S1: Hysteresis Loops for the NiFe films of 20 nm and 40 nm thickness: The orientation of the films was parallel to the field (In-plane configuration). The difference between both Ms values is small, and considering the 10% uncertainty discussed previously, the average value of Ms of 5.4 x 10^5 Am^{-1} was used throughout the work for all NiFe layers.

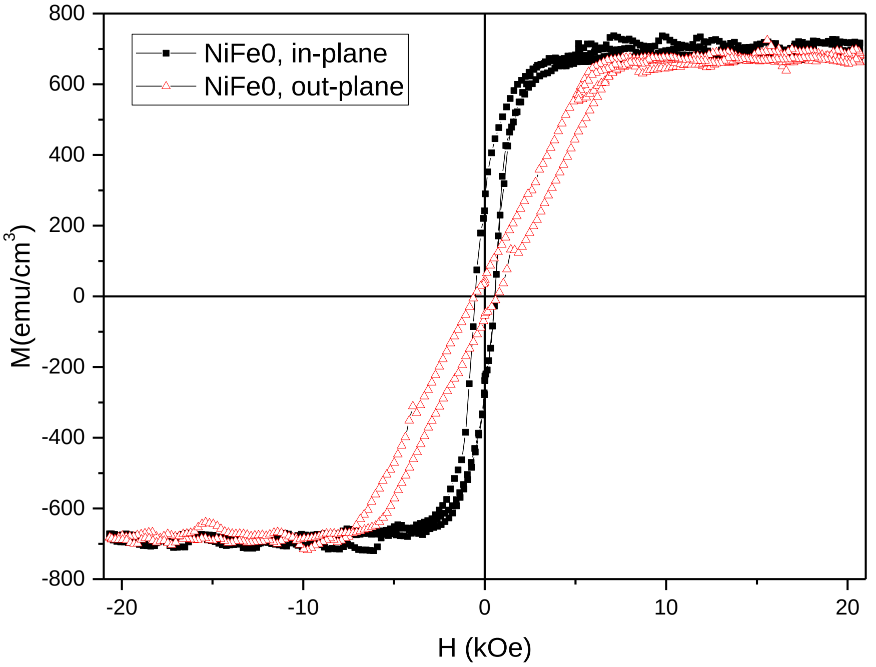

Supplementary Fig. S2: Magnetization curve for NiFe0 sample. [Co0.8/Pd0.8]x50.without NiFe present. For this sample Ms was estimated at 6.8 x 10^5 Am^{-1} and Hs at 7.5kOe.

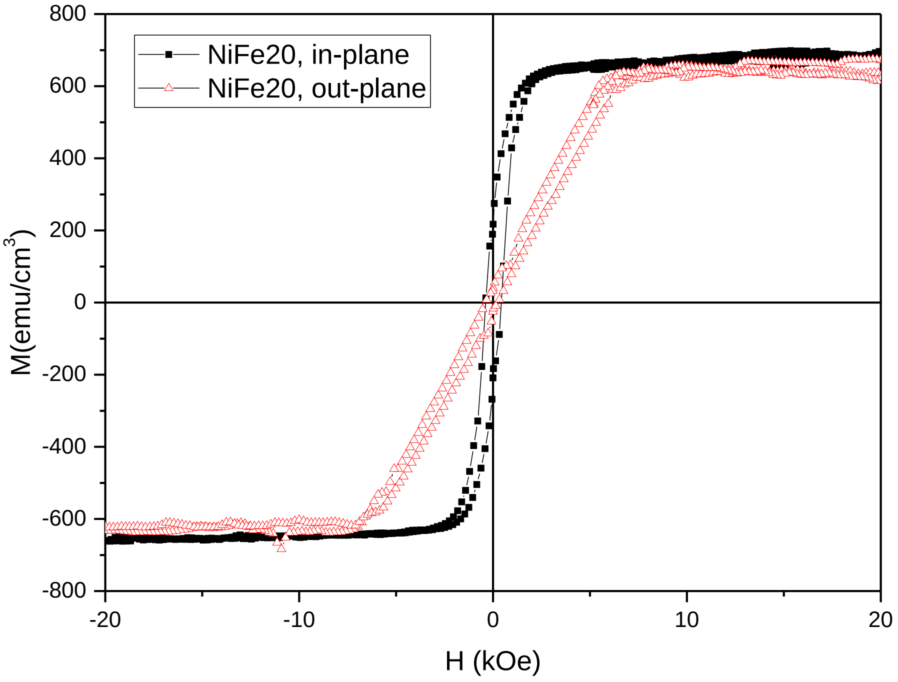

Supplementary Fig. S3: Magnetization curve for NiFe20 sample. For this sample Ms was estimated at 6.5 x 10^5 Am^{-1} and Hs at 7.2kOe.

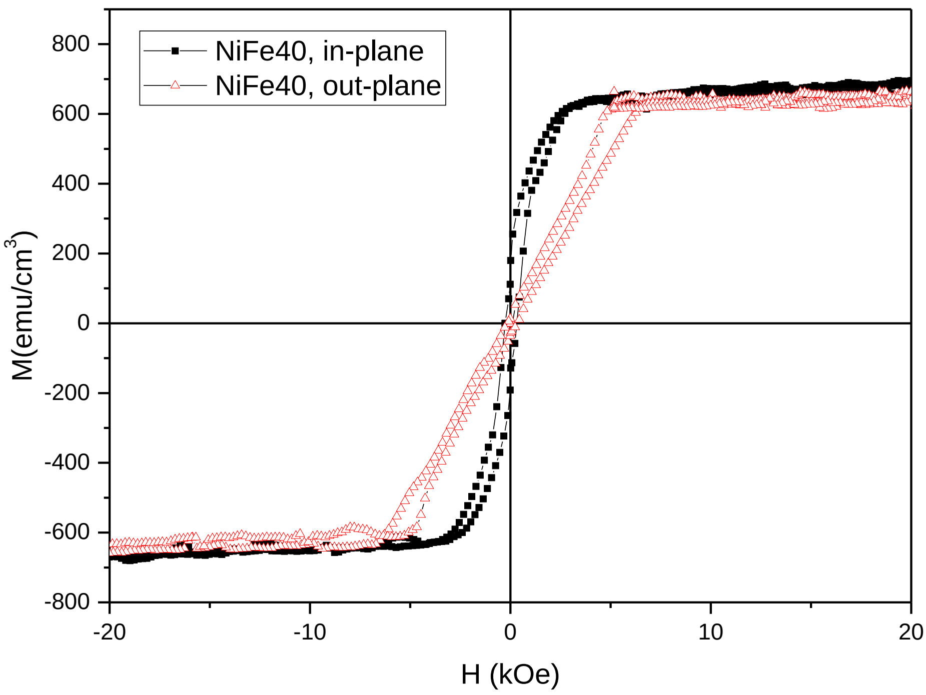

Supplementary Fig. S4: Magnetization curve for NiFe40 sample. For this sample Ms was estimated at 6.5 x 10^5 Am^{-1} and Hs at 6.8kOe.

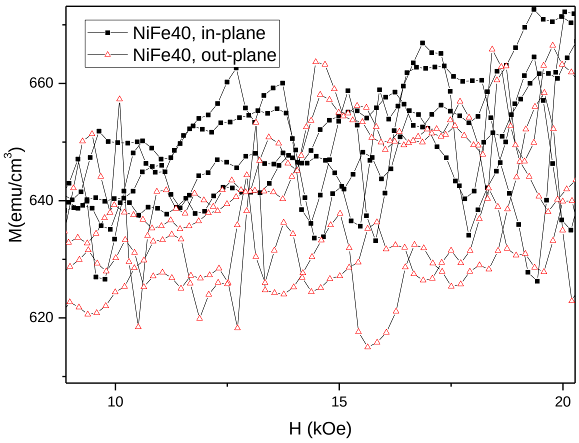

Supplementary Fig S5: Zoom-in of the saturated region of the hysteresis loops for the NiFe40 sample, showing the portion measurement uncertainty of Ms due to noise.
